# Do extra-pulmonary triggers or autonomic neural activity affect rhythm control by anti-arrhythmic drugs in patients with post-ablation atrial fibrillation recurrence?

**DOI:** 10.3389/fcvm.2024.1426531

**Published:** 2024-10-07

**Authors:** Hanjin Park, Hee Tae Yu, Daehoon Kim, Je-Wook Park, Tae-Hoon Kim, Jae-Sun Uhm, Boyoung Joung, Moon-Hyoung Lee, Chun Hwang, Hui-Nam Pak

**Affiliations:** Division of Cardiology, Department of Internal Medicine, Yonsei University College of Medicine, Yonsei University Health System, Seoul, Republic of Korea

**Keywords:** atrial fibrillation, catheter ablation, antiarrhythmic drugs, extra-pulmonary vein trigger, heart rate variability

## Abstract

**Background:**

The role of anti-arrhythmic drugs (AADs) in recurrent atrial fibrillation (AF) after catheter ablation (CA) is not fully understood. The aim of this study was to explore the effects of AADs in patients who recurred after AFCA depending on extra-pulmonary vein triggers (ExPVTs) and post-ablation heart rate variability (HRV) parameters.

**Methods:**

We analyzed 2,036 patients who underwent de-novo AFCA and 486 patients with post-AFCA recurrence who underwent rhythm control with AADs. We investigated the effects of ExPVTs and 3rd month HRV parameters on the post-AFCA recurrence and subsequent AAD responsiveness.

**Results:**

A total of 486 out of 2,036 patients developed clinical recurrence of AF and subsequently underwent rhythm control with AADs. 486 out of 310 patients (63.8%) remained free of second recurrence at 1-year. Post-AFCA recurrence was significantly higher in patients with ExPVT [Log-rank *p* < 0.001, HR 1.45 (1.16–1.83), *p* = 0.001] or higher 3rd month root mean square of the differences between successive RR intervals (rMSSD) [Log-rank *p* < 0.001, HR 1.36 (1.11–1.65), *p* = 0.003] than their counterparts. Patients with ExPVTs during the de-novo procedure had significantly higher 3rd month rMSSD (15.0 [11.0–23.0] vs. 17.0 [11.0–28.0], *p* = 0.022). Patients with high 3rd month rMSSD had higher rate of ExPVTs during the repeat procedure (*n* = 160, 41.0% vs. 22.2%, *p* = 0.019). Among patients with recurrent AF after AFCA, post-AAD recurrence did not differ depending on the presence of ExPVT [Log-rank *p* = 0.455, HR 1.12 (0.78–1.69), *p* = 0.436] or 3rd month rMSSD [Log-rank *p* = 0.457, HR 1.16 (0.87–1.55), *p* = 0.300]. Post-AAD recurrence did not differ between class I_C_ and III AADs (*p* for interaction = 0.311).

**Conclusions:**

ExPVT and post-procedural high rMSSD are independent risk factors for post-AFCA recurrence but not for AAD response in patients with recurrent AF. AADs may suppress ExPVTs and modulate cardiac autonomic activity after post-AFCA recurrence.

## Introduction

Atrial fibrillation (AF) is a global health burden that is associated with substantial morbidity and mortality (1). AF catheter ablation (AFCA) is effective for maintaining sinus rhythm and improving the quality of life in patients with AF (2, 3). However, AF is a progressive disease, and approximately 50% of patients will suffer from recurrent AF and require subsequent rhythm control with either anti-arrhythmic drugs (AADs) or redo-AFCA (3, 4). Despite the utility of redo-AFCA in patients with recurrent AF (5, 6), only a minority of patients will undergo a repeat procedure because of significant economic burden, comorbidities, concerns for complications, or personal preferences (7). This is particularly true in South Korea, where the medical insurance does not cover all redo-AFCAs, and AADs are frequently used as an alternative (to redo-AFCA) when the AF burden or the related symptoms are not substantial. Therefore, understanding the role of AADs in post-AFCA recurrences is of clinical importance.

AADs may exhibit anti-AF effects by suppressing residual PV conduction gaps, extra-PV triggers (ExPVTs) or by modifying the atrial myocardial substrate (8). Their responses are expected to be better in post-AFCA recurrence than they are before the procedure because AFCA significantly reduces AF burden and atrial critical mass (9). Although previous studies report that the mechanism of post-AFCA recurrence is mainly PV reconnection (10, 11), ExPVTs and cardiac autonomic neural imbalance are also important contributors.

ExPVTs were found to be more common in repeat procedures than they are in the de-novo procedures (12) and may play an important role in the recurrence of AF via progression of atrial myopathy, especially in patients with persistent AF (12). In addition, cardiac autonomic neural imbalance is associated with higher recurrence after cardioversion (13) and AADs have vagolytic and beta-blocking properties that may be associated with cardiac autonomic neural activity (14–16).

In this study, we investigated the effects of AADs among patients with restored sinus rhythm after post-AFCA recurrence, and the association with ExPVTs and heart rate variability (HRV) parameters.

## Materials and methods

### Study population

**Figure 1:**
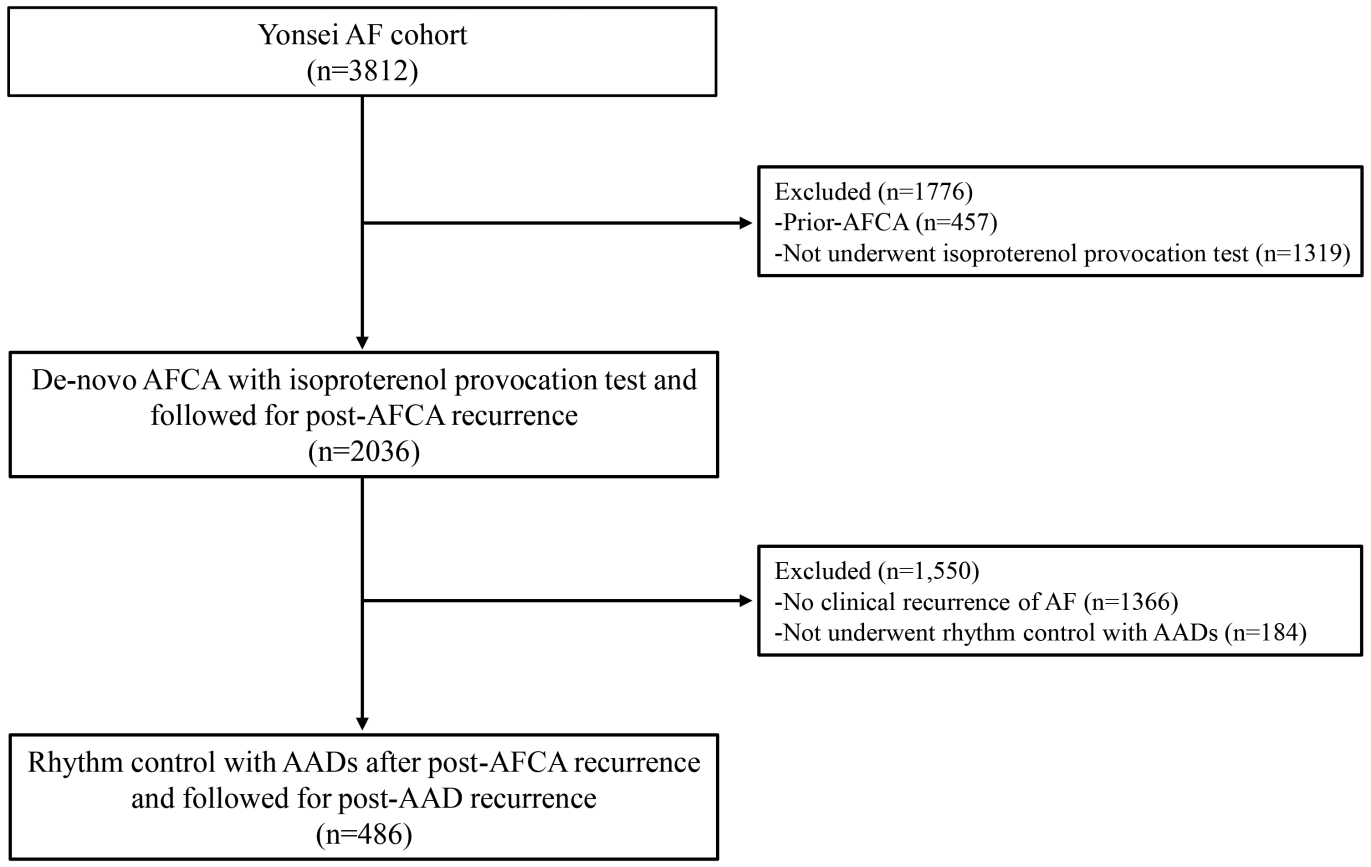
Flow chart of the study population. Among the 3,812 participants in the Yonsei AF cohort, 2,036 participants underwent de-novo AFCA with isoproterenol provocation test and were followed for post-AFCA recurrence. Among the 2,036 participants, 670 participants had post-AFCA recurrence and 486 participants subsequently underwent rhythm control with AADs. AAD, anti-arrhythmic drug; AF, atrial fibrillation; AFCA, atrial fibrillation catheter ablation.

The study protocol adhered to the principles of the Declaration of Helsinki. This study was approved by the Institutional Review Board of the Yonsei University Health System. All patients provided written informed consent for inclusion in the Yonsei AF ablation Cohort Database (URL: https://www.clinicaltrials.gov; unique identifier: NCT02138695). Among the 3,812 participants in the Yonsei AF ablation cohort, we excluded participants who had (1) prior-AFCA (*n* = 457) and (2) not underwent post-procedural isoproterenol provocation test (*n* = 1,319). Finally, a total of 2,036 participants who underwent de-novo AFCA with isoproterenol provocation test between March 2019 and December 2019 were analyzed and followed through December 2021 (Figure 1).

### Echocardiographic evaluation

All participants underwent transthoracic echocardiography before the de-novo procedure. The left atrium volume index (LAVI), left ventricle (LV) ejection fraction, and peak transmitral inflow velocity to tissue doppler echocardiography of the peak septal mitral annular velocity (E/e’) were obtained in accordance with the American Society of Echocardiography guidelines (17).

### Electrophysiological mapping and de-novo AFCA

Antiarrhythmic drugs (AADs) were discontinued more than five half-lives before the procedure Intracardiac electrograms were recorded using the Prucka CardioLab Electrophysiology system (General Electric Medical Systems, Inc, Milwaukee, WI). Multiview pulmonary venograms were obtained following a trans-septal puncture. Intravenous heparin was used to maintain an activated clotting time of 350 to 400 s during the procedure. AFCA was performed in all patients using a 3-dimensional electroanatomic mapping system (NavX; Abbott, Inc, Minnetonka, MN) merged with 3-dimensional spiral computed tomography. We used an open irrigated, 3.5 mm tip deflectable catheter (Celsius and Smart-Touch; Johnson & Johnson, Inc., Diamond Bar, CA; Coolflex and FlexAbility, Abbott, Inc., Minnetonka, MN) with radiofrequency (RF) power of 30–60 W at the anterior part of the LA for 30 s and RF power of 20–50W at the posterior part of the LA and PVs for <30s.

All patients underwent circumferential pulmonary vein isolation (CPVI). CPVI was defined as confirmation of bidirectional block and electrical isolation of the PVs after creation of a circumferential, continuous lesion at the LA antrum that encircled the right and light PVs. Majority of patients with a previously documented ECG of cavotricuspid isthmus dependent (CTI) atrial flutter underwent CTI ablation.

Additional empirical linear ablation was performed at the operator's discretion among patients with persistent or long-standing persistent AF. Additional empirical linear ablation included extra PV LA linear lines (e.g., posteroinferior line, roof line, anterior line, and left lateral isthmus line) and extra PV RA linear lines (e,g, CTI line, and superior vena cava-right atrial septal line), similar to that of the Cox Maze III lesion set based on the mechanistic ground that AF is less likely to persistent in small, segmented atria (19, 20). Despite uncertain benefits beyond CPVI, additional empirical linear ablation remains a useful ablation strategy employed by 10 to 25% of the writing group in the expert consensus on statement of catheter or surgical ablation of AF (18, 19).

### Isoproterenol provocation and procedural endpoints

After completion of the CPVI or extra-PV linear ablation, AF or atrial tachycardia (AT) was induced by 10 s high-current burst pacing from the right atrial (RA) electrodes. A pacing cycle length was started at 250 ms and gradually reduced to 120 ms (21). Isoproterenol (5–20 μg/min depending on the use of beta-blockers with a target heart rate of 120 bpm) was administered for at least 3 min before and after the induction of AF or AT. This protocol, which involved aggressive atrial burst pacing than that previously reported (22), was to achieve Ca^2+^ overload by rapid atrial pacing or induction of AF, and subsequent electrical cardioversion that might reveal an extra-PV trigger. Details of the isoproterenol provocation protocol is provided elsewhere (21, 23–26). In the case of sustained AF or AT, a synchronized internal cardioversion was applied via a biphasic shock of 2–20J using high right atrial catheter serving as the cathode and the coronary sinus catheter as the anode for shock delivery. We chose internal cardioversion because internal cardioversion is possible under conscious sedation and also not affected by tissue resistance (e.g., obese patients). The procedure was ended in case of negative ExPVT. A negative ExPVT was defined as absence of immediate recurrence of AF originating from non-PV foci or post-procedural atrial premature complexes (APCs) of less than 6 beats per minute during the isoproterenol provocation after successful electrical cardioversion (21). When further ExPVTs were observed while maintaining isoproterenol infusion, we determined the location of ExPVTs based on contact bipolar electrograms and mapped the ExPVTs using a 3D-activation mapping with a multielectrode catheter. A positive ExPVT was defined as AF triggers originating from non-PV foci or APCs of 6 or more beats per minute during the isoproterenol provocation (12). Based on the bipolar electrograms and 3D activation mapping of the ExPVTs, we carefully ablated the earliest non-PV triggering points (35–50W for 10 s each). After ablation of non-PV triggering points, isoproterenol provocation test was repeated. In case of remaining ExPVT, we repeated ablation of non-PV triggering points until a negative ExPVT was confirmed.

### Follow-up for post-AFCA recurrence

Participants were discharged without AADs except for those who had symptomatic frequent APC, non-sustained AT, or early recurrence of AF on telemetry monitoring during the admission period (*n* = 407). Patients with early recurrence were discharged with AADs, despite within the blanking period (19), because early recurrence is a well-established risk marker for clinical recurrence (27).

After discharge, patients were regularly followed up at the outpatient clinic (1, 3, 6, and 12 months from discharge and every 6 months thereafter) or whenever the patient experienced symptoms suggestive of AF recurrence. We obtained an electrocardiogram (ECG) at every outpatient visit and Holter ECG recordings (24-hour) were obtained at 3 and 6 months, and every 6 months thereafter according to the 2017 Heart Rhythm Society/European Heart Rhythm Association/European Cardiac Arrhythmia Society Expert Consensus Statement guidelines (28). Patients who experienced symptoms suggestive of an arrhythmia recurrence were additionally evaluated using a Holter ECG monitor or event monitor recording. A researcher whose assignment was independent of the study group conducted the Holter ECG analysis and adjudication. Post-AFCA recurrence was defined as any episode of AF or AT of at least 30 s. Any ECG documentation of AF recurrence less than 3 months after the procedure was considered an early recurrence and an AF recurrence more than 3 months after the procedure was considered a clinical recurrence.

### Post-AFCA HRV parameters

The post-procedural HRV parameters were obtained via 24-h Holter ECG monitor recordings 3 months after the procedure with a GE Marquette MARS 8000 Holter analyzer (General Electric Medical System, Inc.). Only high-quality recordings were selected for the analysis. All recordings were digitalized and reviewed by an experienced operator. Among the 2,036 participants, 1,293 participants had available post-procedural HRV parameters. The reasons for missing post-procedural HRV data include low quality recordings, electrical artifacts, frequent APCs or premature ventricular complexes, underwent 24-hr Holter ECG monitoring exams in another hospital near patients’ residence, or receiving autonomically active drugs such as AADs at the time of Holter monitoring.

The time domain HRV parameters were analyzed as the mean heart rate and root mean square of the differences between successive RR intervals (rMSSD). The frequency domain HRV parameters were analyzed as the low-frequency (LF) components (band of power spectrum range between 0.040 and 0.150 Hz), high-frequency (HF) components (band of power spectrum range between 0.150 and 0.400 Hz), and the LF/HF ratio.

In brief, mean heart rate is a surrogate of HRV. The rMSSD represents the beat-to-beat variance in the heart rate in which a low mean heart rate and high rMSSD indicate an increased HRV (29). The HF component represents parasympathetic nervous activity. The LF component represents sympathetic nervous activity, and the LF/HF ratio represents a sympathovagal balance (30).

### Management for post-AFCA recurrence and subsequent follow-up for post-AAD recurrence

We prescribed guideline-based AADs for patients with post-AFCA recurrence (2). If sinus rhythm was not restored despite the use of AADs, electrical cardioversion was performed. After restoration of sinus rhythm, patients using AADs underwent ECG recordings during every visit, as well as regular 24-h Holter ECG monitor recording in the outpatient clinic for post-AAD recurrence. Post-AAD recurrence was defined as any episode of AF or AT that lasted for at least 30 s. The rhythm follow-up for post-AAD recurrence was the same as that for post-AFCA recurrence.

Patients who had post-AFCA recurrence but were not treated with AADs for rhythm control had the following reasons: underlying bradyarrhythmia, immediate redo-AFCA without AAD use, side effects with AAD use, or patient preference who had no AF-related symptoms after recurrence.

### Electrophysiological mapping and redo-AFCA

CPVI was checked for all patients during the redo procedure. CPVI ablation and bidirectional block was again achieved in case of PV reconnections. For patents who underwent additional linear ablations at the de-novo procedure, we checked whether there were any reconnections at the linear lines. Further linear line ablation and bidirectional block was achieved in case of reconnections at the linear lines. After enforcement of the de-novo ablation sites, ExPVTs was checked using the same isoproterenol provocation as described earlier. In case of positive ExPVTs, we carefully mapped and ablated any non-PV triggering points (31).

### Statistical analysis

Categorical variables are reported as numbers (percentages). Categorical variables were analyzed using the Chi-square or Fisher's exact test. Continuous variables were examined to investigate a normal distribution using the Shapiro-Wilk or Kolmogorov-Smirnov tests. Continuous variables with a normal distribution are reported as means ± standard deviation and those without a normal distribution are reported as medians and interquartile ranges. The ANOVA test was used to compare the continuous variables with a normal distribution. The Kruskal-Wallis test was used to compare the continuous variables without a normal distribution.

We conducted a Kaplan–Meier analysis with a log-rank test to compare the freedom from post-AFCA and post-AAD recurrence according to ExPVT and HRVs. The cut-off values for the high and low levels of HRV parameters were derived from the Youden index. In addition, we conducted a Cox proportional hazards regression analysis to investigate the predictors associated with post-AFCA and post-AAD recurrence. The proportional hazard assumption was not violated, as examined by Schoenfeld residual plots (32). We performed multiple subgroup analyses using the Cox proportional hazards regression analysis and the specified baseline covariates to detect any potential interaction with post-AFCA and post-AAD recurrence. In addition, because additional linear ablations after CPVI might affect ExPVTs and HRV parameters, we repeated the main analysis after excluding those who underwent extra PV LA ablation during the de-novo procedure.

All analyses were performed using R statistics, version 4.0.2 software (R Foundation for Statistical Computing), and a two-sided *p*-value < 0.05 was considered statistically significant.

## Results

### Baseline characteristics of the study population followed for post-AFCA recurrence

A total of 2,036 patients underwent de-novo AFCA, and 32.9% (670 of 2,036) of them experienced post-AFCA recurrences. The baseline characteristics of the study participants are presented in Table 1. In brief, the study participants were median 59 (52–67) years, 573 (28.1%) were female, and 1,373 (67.4%) had paroxysmal AF. Participants with ExPVTs [12.4% (253 of 2,036)] were older (*p* = 0.009), likely to be female (*p* = 0.006), and undergo more extra-PV LA ablations (*p* < 0.001) than those without. The location of the ExPVTs identified are presented in Supplementary Table 1. We also present the type of AADs used at pre-ablation in Supplementary Table 2.

**Table 1:** Baseline characteristics of the study population at the time of de-novo AFCA according to extra-PV triggers.

	De-novo AFCA			
	Overall	Extra-PV trigger (-)	Extra-PV trigger (+)	*p*-value
Total, n	2,036	1,783	253	
Age, years	59 (52–67)	59 (51–67)	61 (54–68)	0.009
Female	573 (28.1)	483 (27.1)	90 (35.6)	0.006
Paroxysmal AF	1,373 (67.4)	1,209 (67.8)	164 (64.8)	0.381
BMI, kg/m^2^	24.7 (22.9–26.6)	24.7 (22.9–26.7)	24.4 (22.7–26.4)	0.180
Comorbidity				
HTN	915 (44.9)	799 (44.8)	116 (45.8)	0.808
DM	291 (14.3)	258 (14.5)	33 (13.0)	0.610
Heart failure	271 (13.3)	233 (13.1)	38 (15.0)	0.449
Vascular disease	222 (10.9)	205 (11.5)	17 (6.7)	0.030
Echocardiography parameter				
EF,%	64.0 (59.0–68.0)	64.0 (59.0–69.0)	64.0 (60.0–68.0)	0.903
E/e’	9.0 (7.4–12.0)	9.0 (7.3–12.0)	9.4 (8.0–12.0)	0.098
LAVI, ml/m^2^	34.5 (27.5	34.0 (27.3–42.9)	37.4 (29.6–48.9)	<0.001
Empirical linear ablation				
Extra-PV LA linear line	780 (38.3)	648 (36.3)	132 (52.2)	<0.001
Extra-PV RA linear line	1,489 (73.1)	1,274 (71.5)	215 (85.0)	<0.001
Medication				
RAAS inhibitors	697 (34.3)	605 (34.0)	92 (36.4)	0.500
Beta blockers	624 (30.7)	553 (31.1)	71 (28.1)	0.370
Heart rate variability (*n* = 1,293)				
Mean heart rate, bpm	74 (66–82)	74 (67–82)	71 (63–80)	<0.001
rMSSD, ms	15.0 (11.0–23.0)	15.0 (11.0–23.0)	17.0 (11.0–28.0)	0.022
LF, Hz	5.7 (3.4–10.2)	5.7 (3.4–9.9)	5.9 (3.3–11.6)	0.215
HF, Hz	5.3 (3.8–8.3)	5.3 (3.8–8.2)	5.9 (4.0–10.8)	0.075
LF/HF ratio	1.0 (0.8–1.4)	1.0 (0.8–1.4)	1.0 (0.8–1.4)	0.916

AF, atrial fibrillation; AFCA, atrial fibrillation catheter ablation; BMI, body mass index; DM, diabetes mellitus; E/e’, ratio of early diastolic mitral inflow velocity to early diastolic mitral annulus velocity; HF, high frequency; HTN, hypertension; EF, ejection fraction; LA, left atrium; LAVI, left atrial volume index; LF, low frequency; PV, pulmonary vein; RA, right atrium; rMSSD, root mean square of differences between successive RR intervals.

### Effects of ExPVT and rMSSD on the rhythm outcome after AFCA

**Figure 2:**
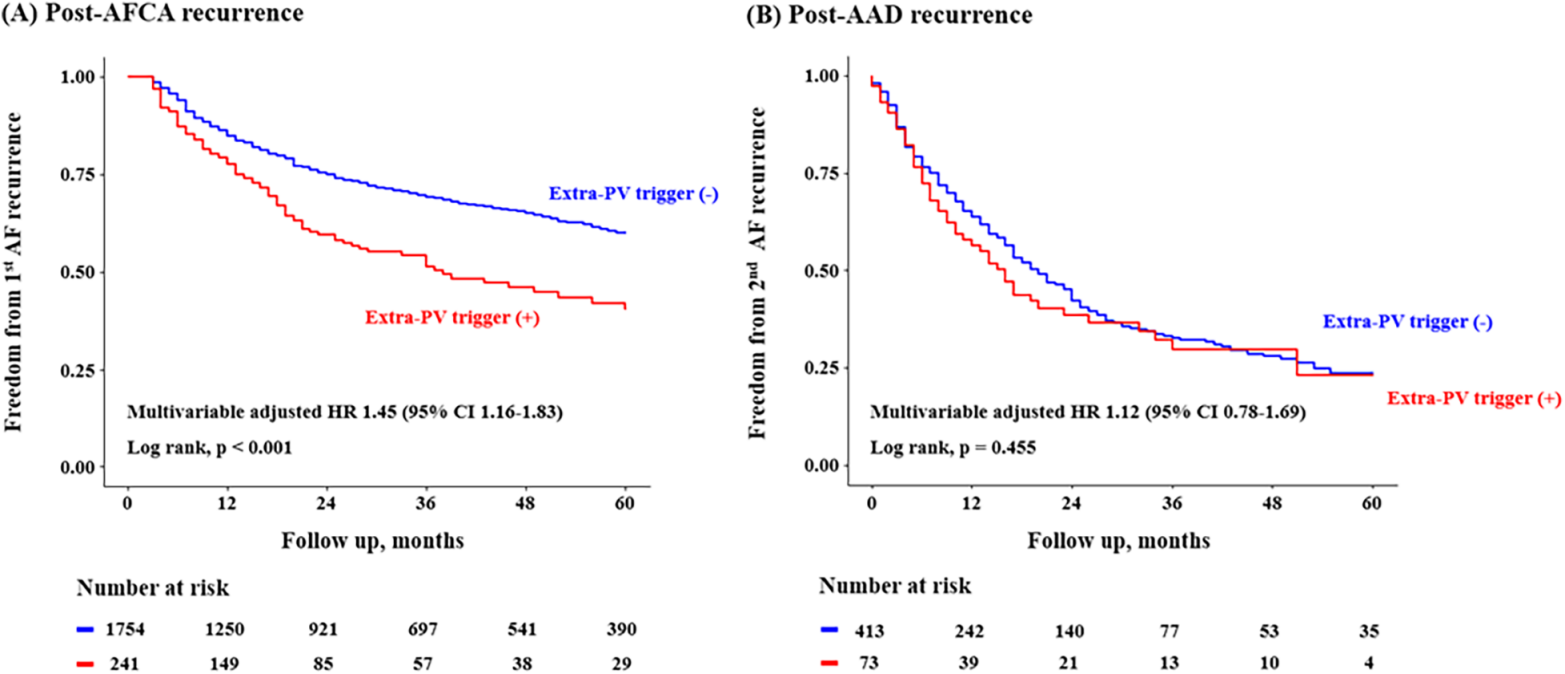
Freedom from post-AFCA recurrence and subsequent AAD responsiveness according to extra-PV triggers. Kaplan-Meier analysis and Cox proportional hazard regression analysis for the post-AFCA and post-AAD recurrence according to extra-PV triggers is presented. Covariates adjusted for the Cox proportional hazard model was the same as in Table 2. AAD, anti-arrhythmic drug; AFCA, atrial fibrillation catheter ablation; CI, confidence interval; HR, hazard ratio; PV, pulmonary vein.

**Figure 3:**
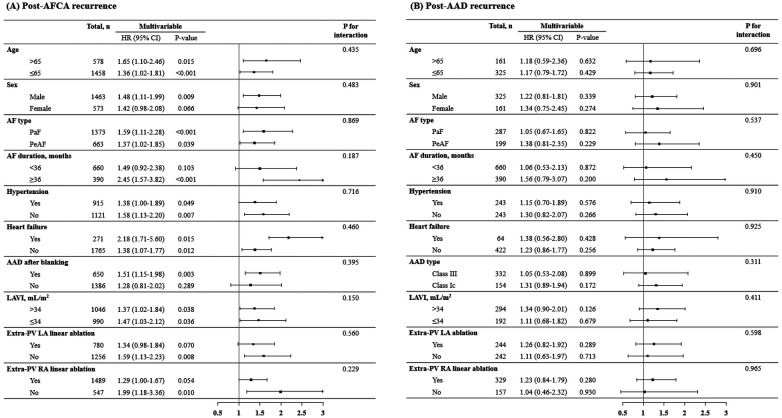
Subgroup analysis of post-AFCA recurrence and subsequent AAD responsiveness according to extra-PV triggers. HRs for the risk of post-AFCA and post-AAD recurrence among patients with extra-PV triggers compared to those without stratified by multiple subgroups are presented. Covariates adjusted for the Cox proportional hazard model was the same as in Table 2. AAD, anti-arrhythmic drug; AF, atrial fibrillation; AFCA, atrial fibrillation catheter ablation; CI, confidence interval; HR, hazard ratio; LAVI, left atrial volume index; PaF, paroxysmal atrial fibrillation; PeAF, persistent atrial fibrillation; PV, pulmonary vein; RA, right atrium.

In the Kaplan-Meier analysis, the rate of post-AFCA recurrence was significantly higher among patients with ExPVTs (Log rank, *p* < 0.001, Figure 2). Similarly in the Cox regression analysis, the risk of post-AFCA recurrence was significantly higher among patients with ExPVTs (adjusted hazard ratio [HR] 1.45, 95% confidence interval [CI] 1.16–1.83, *p* = 0.001, Table 2). These associations were independent of age, sex, AF type, AF duration, heart failure, or AAD use after 3 months of the blanking period (*p* for interaction >0.05 for all specified covariates, Figure 3).

**Table 2:** Risk of post-AFCA recurrence and subsequent AAD responsiveness according to extra-PV trigger and HRVs.

	Post-AFCA recurrence				Post-AAD recurrence			
	Univariable		Multivariable^a^		Univariable		Multivariable^a^	
	HR (95% CI)	*P*-value	HR (95% CI)	*P*-value	HR (95% CI)	*P*-value	HR (95% CI)	*P*-value
Extra-PV trigger	1.81 (1.46–2.24)	<0.001	1.45 (1.16–1.83)	0.001	1.12 (0.83–1.52)	0.456	1.12 (0.78–1.69)	0.436
HRV parameters^b^								
Low mean heart rate	0.62 (0.52–0.74)	<0.001	0.75 (0.62–0.92)	0.005	0.77 (0.60–0.99)	0.045	0.86 (0.64–1.15)	0.305
High rMSSD	1.63 (1.37–1.95)	<0.001	1.36 (1.11–1.65)	0.003	1.23 (0.95–1.59)	0.109	1.16 (0.87–1.55)	0.300
High LF	1.60 (1.34–1.91)	<0.001	1.41 (1.16–1.71)	<0.001	1.01 (0.78–1.30)	0.961	0.97 (0.73–1.28)	0.810
High HF	1.69 (1.41–2.02)	<0.001	1.43 (1.17–1.74)	<0.001	1.05 (0.81–1.36)	0.699	1.01 (0.75–1.34)	0.967
High LF/HF ratio	1.23 (1.00–1.50)	0.048	1.25 (1.00–1.56)	0.049	1.04 (0.77–1.42)	0.792	0.97 (0.69–1.35)	0.857

AAD, anti-arrhythmic drug; AF, atrial fibrillation; AFCA, atrial fibrillation catheter ablation; BMI, body mass index; CI, confidence interval; DM, diabetes mellitus; E/e’, ratio of early diastolic mitral inflow velocity to early diastolic mitral annulus velocity; HR, hazard ratio; HRV, heart rate variability; HF, high frequency; HTN, hypertension; EF, ejection fraction; LAVI, left atrial volume index; LF, low frequency; PV, pulmonary vein; rMSSD, root mean square of differences between successive RR intervals.

^a^ Multivariable Cox proportional hazard model was adjusted for age, sex, AF type, HTN, DM, BMI, heart failure, vascular disease, EF, E/e’, LAVI, Extra-PV LA linear ablation, Extra-PV RA linear ablation, and AAD after 3 months of a blanking period.

^b^ Cut-off values for HRV parameters were derived from the Youden index.

**Figure 4:**
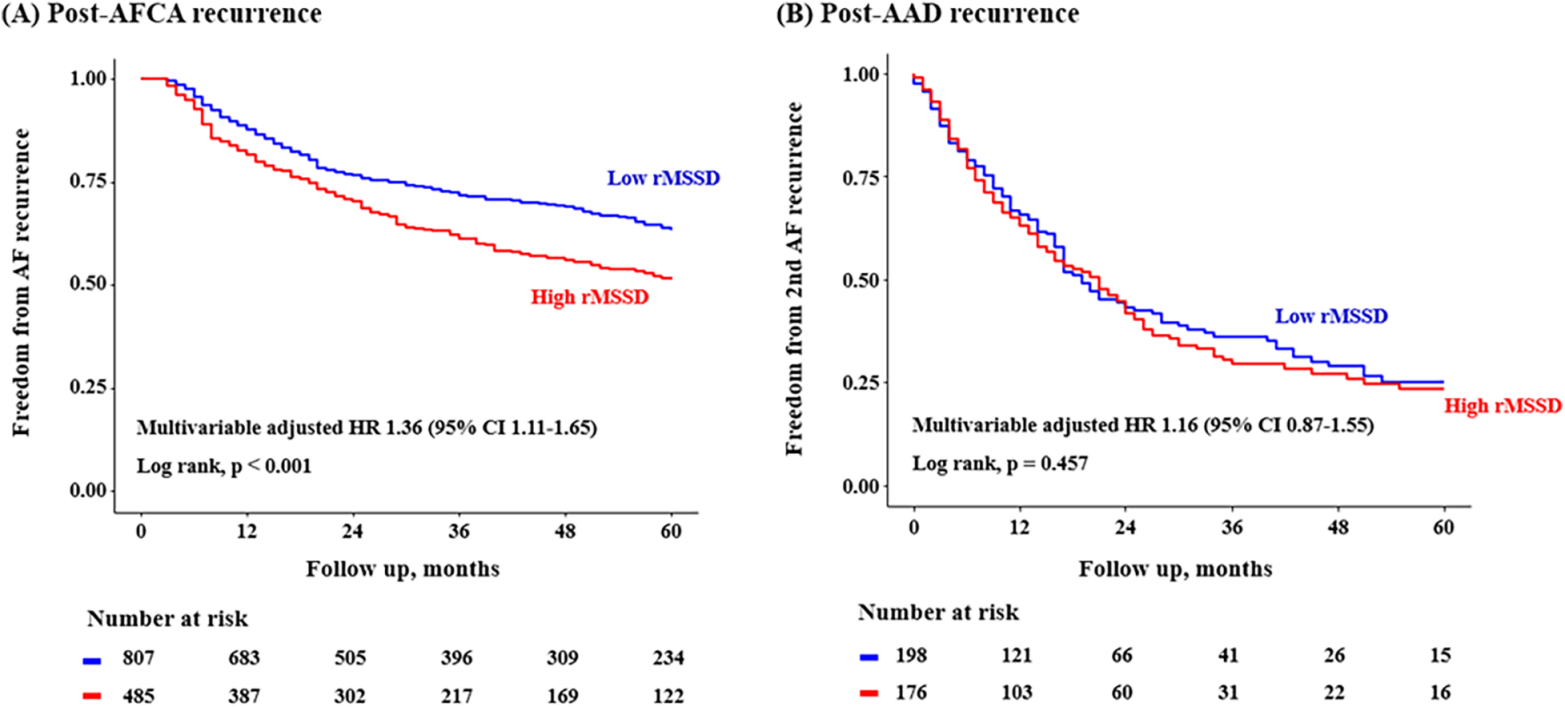
Freedom from post-AFCA recurrence and subsequent AAD responsiveness according to rMSSD. HRs for the risk of post-AFCA and post-AAD recurrence among patients with high rMSSD compared to those with low rMSSD stratified by multiple subgroups are presented. Covariates adjusted for the Cox proportional hazard model was the same as in Table 2. The cut-off value for rMSSD was derived from the Youden index. AAD, anti-arrhythmic drug; AFCA, atrial fibrillation catheter ablation; CI, confidence interval; HR, hazard ratio; rMSSD, root mean square of differences between successive RR intervals.

Post-AFCA recurrence was assessed according to the level of post-procedural HRV parameters. A total of 1,293 patients with available post-procedural HRV data were analyzed. In the Kaplan-Meier analysis, the rate of post-AFCA recurrence was significantly higher among patients with high 3rd month rMSSD (Log rank, *p* < 0.001, Figure 4). Similarly in the Cox regression analysis, the risk of post-AFCA recurrence was significantly higher among patients with high 3rd month rMSSD (adjusted HR 1.36, 95% CI 1.11–1.65, *p* = 0.003). Similar trend was found for other HRV parameters including mean heart rate, LF, HF, and LF/HF ratio (Supplementary Figure 1). The effects of ExPVT and rMSSD on the post-AFCA recurrence remained consistent after excluding those who underwent extra PV LA ablation (Supplementary Figure 2).

### Association between ExPVT and rMSSD

**Figure 5:**
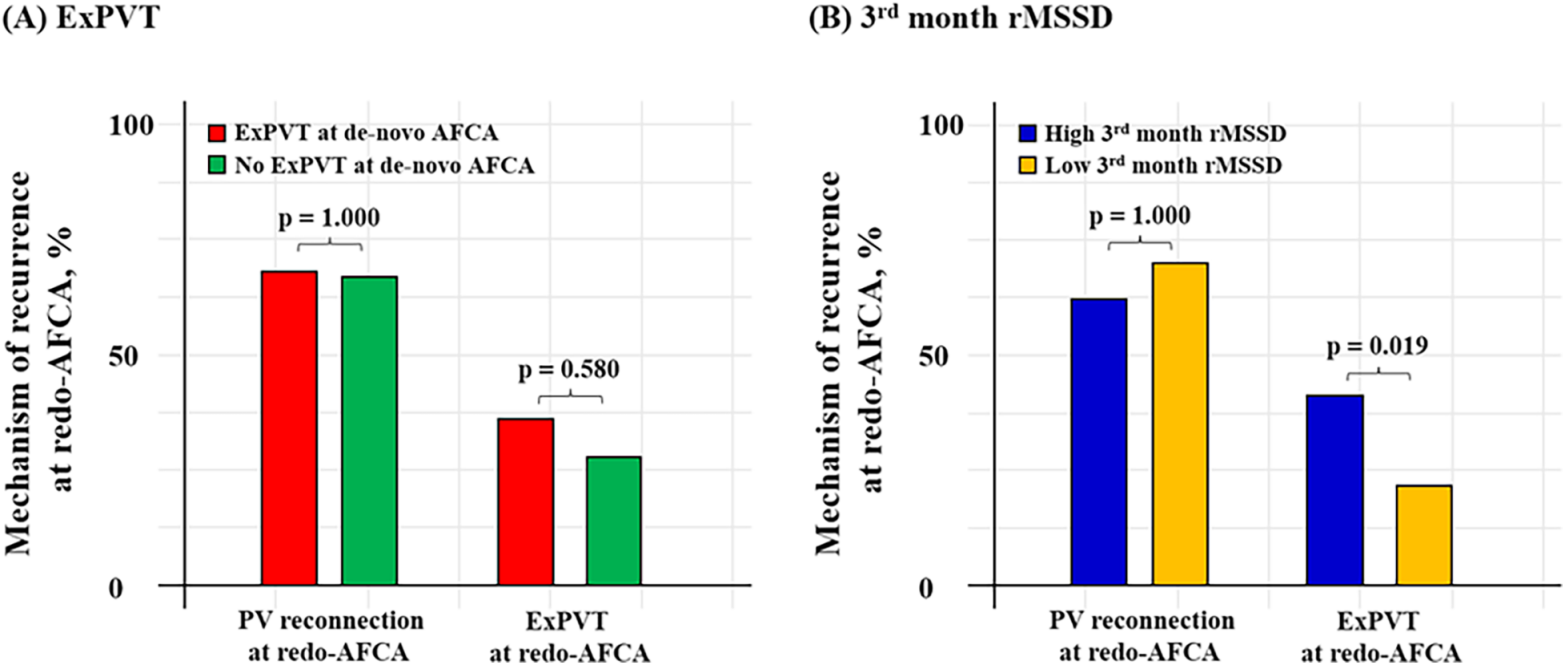
Mechanism of 2nd recurrence by ExPVT and 3rd month rMSSD. Mechanism of recurrence identified at the repeat-AFCA according to ExPVT and 3rd month rMSSD is presented as bar graphs. Patients with high 3rd month rMSSD after the de-novo AFCA showed higher rate of ExPVT as the mechanism of recurrence. The cut-off value for rMSSD was derived from the Youden index. AFCA, atrial fibrillation catheter ablation; ExPVT, extra pulmonary vein triggers; rMSSD, root mean square of differences between successive RR intervals; PV, pulmonary vein.

Patients with ExPVTs had significantly higher 3rd month rMSSD [17.0 (11.0–28.0) with ExPVTs vs. 15.0 (11.0–23.0) without ExPVTs, *p* = 0.022]. In addition, patients with high 3rd month rMSSD showed significantly higher rate of ExPVTs as the mechanism of recurrence (ExPVT; 41.0% for high 3rd month rMSSD vs. 22.2% for low 3rd month rMSSD, *p* = 0.019, Figure 5).

### Baseline characteristics of the study population followed for post-AAD recurrence

Among the 760 patients with post-AFCA recurrence, 72.5% (486 of 670) subsequently underwent rhythm control with AADs. 63.8% (310 of 486) remained free of post-AAD recurrence after 1 year (66.6% [191 of 287] for paroxysmal AF and 59.8% [119 of 199] for persistent AF). The baseline characteristics are presented in Supplementary Table 3. The rate of post-AAD recurrence was not significantly different according to the type of AAD used after post-AFCA recurrence (Supplementary Table 4). The type of AADs used did not significantly differ according to the presence of ExPVTs (*p* = 0.220).

### AAD effects depending on ExPVT or rMSSD

In the Kaplan-Meier analysis, the rate of post-AAD recurrence was not significantly different depending on ExPVTs (Log-rank, *p* = 0.455, Figure 2). Similarly in the Cox regression analysis, the risk of post-AAD recurrence did not significantly differ depending on ExPVTs (adjusted HR 1.12, 95% CI 0.78–1.69, *p* = 0.436, Table 2). The presence of ExPVTs was not associated with a higher risk of post-AAD recurrence in any of the specified subgroups including use of either class Ic or III AADs (*p* for interaction >0.05 for all specified covariates, Figure 3).

In addition, post-AAD recurrence was not significantly different according to the time-domain (Log-rank, *p* = 0.457 for rMSSD, Figure 4, and *p* = 0.183 for mean heart rate, Supplementary Figure 1) and frequency-domain HRVs (Log-rank, *p* = 0.959, 0.684, and 0.857 for LF, HF, and LF/HF ratio, respectively, Supplementary Figure 1). The risk of post-AFCA and post-AAD recurrence according to other baseline covariates is presented in Supplementary Table 5. The effects of ExPVT and rMSSD on the post-AAD recurrence after excluding those who underwent extra PV LA ablation are presented in Supplementary Figure 2. The results and trends were similar with the main analysis.

## Discussion

In this retrospective analysis of a single-center cohort, we found that ExPVTs and high post-procedural 3rd month rMSSD were independent risk factors for post-AFCA recurrence. However, these associations were not significant among patients with post-AFCA recurrence who subsequently underwent rhythm control with AADs. ExPVTs were associated with significantly higher 3rd month rMSSD and patients with high 3rd month rMSSD were found to have ExPVTs as the dominant mechanism of recurrence without significant difference in PV reconnection rates identified at the redo procedure (Figure 5). These findings suggest that ExPVTs and high rMSSD are interrelated conditions and that AADs may play a role in suppressing ExPVTs and modulating cardiac autonomic neural activity in patients with post-AFCA recurrence.

### Rhythm control after post-AFCA recurrence

Several studies compared the efficacy of redo-AFCA vs. medical therapy in post-AFCA recurrences (33–36). A randomized trial from China reported that catheter ablation in recurrent AT after ablation of persistent AF was superior to medical therapy in maintaining sinus rhythm (34). In addition, a prospective cohort study from Germany reported that redo-AFCA is associated with symptomatic relief that is comparable to an index procedure (33). Although redo-AFCA is an efficient rhythm control strategy among patients with post-AFCA recurrence (5, 6), it is only performed in a minority of patients because of financial implications, associated complications, or individual preferences (7). In contrast, AADs represent a cost-effective and readily implementable rhythm control strategy. However, the exact role of AADs in managing patients with post-AFCA recurrence is yet to be determined.

### Mechanisms of post-AFCA recurrence and the role of AADs

Potential mechanisms of post-AFCA recurrence include residual conduction gaps of the isolated PVs, extra-PV triggers, cardiac autonomic neural imbalance, and arrhythmogenic atrial substrate (9). A randomized trial from Belgium reported that continuing AADs after AF catheter ablation reduces the risk of recurrent AF and that AADs might block residual conduction gaps in the LA-PV junction or suppress ExPVTs (8). Consistent with their findings, this study suggests that AADs may suppress ExPVTs in the context of post-AFCA recurrence. The potential mechanism might be associated with anti-AF effects of AADs that promote prolongation of action potential duration, reduction of resting membrane potential as well as conduction velocities (9).

Beyond suppression of ExPVTs, our results also suggest that AADs may have a cardiac autonomic neural modulating activity. Indeed, there were several animal experimental evidences that elucidate the role and mechanism of AADs in cardiac autonomic neural modulation (37, 38). For example, Hohnloser et al. reported that sotalol therapy was associated with significant improvement in indices for parasympathetic tone in patients with ventricular arrhythmia (38). The underlying mechanism might be associated with vagolytic and beta-blocking properties of AADs that affects cardiac sympathetic/parasympathetic nervous activity (14–16, 39). Cardiac autonomic neural imbalance may promote ExPVTs by enhancing automaticity, prolonging the action potential duration, and increasing the delay after depolarization (40). In this study, ExPVTs were associated with high post-procedural rMSSD, and those who had high post-procedural rMSSD exhibited ExPVT as the dominant mechanism for post-AFCA recurrences at the redo procedure. These findings indicate that ExPVT and rMSSD are inter-related conditions and might partially explain the reason for AADs affecting both ExPVTs and cardiac autonomic neural activity

### Repeat procedure vs. AADs for patients with post-AFCA recurrence

The EAST-AFNET 4 trial (41) demonstrated the utility of rhythm control in patients with AF. However, there is limited knowledge of the effects of AADs among patients with post-AFCA recurrence. Among PAF patients with post-AFCA recurrence, 66.6% remained free of post-AAD recurrence after 1 year. This finding was comparable to previous reports on the efficacy of repeat-AFCA among patients with PAF (42, 43). Among persistent AF patients with post-AFCA recurrence, 59.8% remained free of post-AAD recurrence after 1 year, which is comparable to previous reports on the efficacy of repeat-AFCA among patients with persistent AF (44). These findings indicate that AADs may serve as an alternative rhythm control strategy in patients with post-AFCA recurrence. AADs may be especially useful among patients with PAF and those who are not planned for a redo procedure.

## Limitations

There are several limitations to this study. First, this was a single-center study with a limited number of patients. Therefore, the generalizability of our results is limited. However, data from a single-center are still valuable because the AFCA procedure and the rhythm follow-up protocols are largely consistent. Second, this was a case-only study in which there were no controls for comparison (i.e., patients who did not undergo rhythm control with AADs after post-AFCA recurrence). However, it would be unethical not to use AADs in patients with post-AFCA recurrence unless contraindicated. Third, the post-procedural HRV analyses should be interpreted carefully because not all patients had available HRV parameters. Fourth, we included patients with isoproterenol provocations and excluded those who did not undergo such a test. This exclusion may have introduced selection bias. Fifth, there was no uniform strategy for extra-PV LA, and ExPVT ablation and was left to the operator's discretion. Sixth, among the 160 participants who underwent repeat-AFCA, 136 (84.4%) patients had no ExPVTs at the de-novo AFCA and 38 (28.1%) of them subsequently developed ExPVTs identified at the repeat-AFCA, and this group of patients might reflect inner limitation of the protocols for detection of extra-PV triggers (45).

## Conclusions

ExPVT and post-procedural high rMSSD are independent risk factors for post-AFCA recurrence, but not for AAD response in patients with recurrent AF. Further investigation is needed to determine whether AADs suppress ExPVT and modulate cardiac autonomic activity after post-AFCA recurrence.

## Data Availability

The data analyzed in this study is subject to the following licenses/restrictions: the data, analytic methods, and study materials are available from the corresponding author upon reasonable request. Requests to access these datasets should be directed to heetyu@yuhs.ac.
