# Supplementary material for: Do extra-pulmonary triggers or autonomic neural activity affect rhythm control by anti-arrhythmic drugs in patients with post-ablation atrial fibrillation recurrence?
Source: Front Cardiovasc Med. 2024 Oct 7;11:1426531. doi: 10.3389/fcvm.2024.1426531 (PMC11491320; doi:10.3389/fcvm.2024.1426531)
Supplement: Supplementary file 1 [file Datasheet1.docx]

**Supplemental Materials**

**Supplemental Table 1.** Location of the extra-PV triggers at the de-novo and repeat-AFCA.

**Supplemental Table 2**. Type of AAD used at pre-AFCA.

**Supplemental Table 3**. Baseline characteristics of the study population among post-AFCA recurrence who underwent rhythm control with AADs.

**Supplemental Table 4.** AAD responsiveness according to type of AAD used after post-AFCA recurrence.

**Supplemental Table 5**. Risk of post-AFCA recurrence and subsequent AAD responsiveness according to baseline covariates.

**Supplemental Figure 1**. Freedom from post-AFCA recurrence and subsequent AAD responsiveness according to HRV parameters.

**Supplemental Figure 2**. Freedom from post-AFCA recurrence and subsequent AAD responsiveness according to ExPVT and rMSSD after excluding those who underwent extra PV LA ablations.

**Supplemental Table 1.** Location of the extra-PV triggers at the de-novo and repeat-AFCA.

|  | **De-novo AFCA**  **(n=253 out of 2036)** | **Repeat-AFCA**  **(n=47 out of 160)** |
| --- | --- | --- |
| **Extra PV trigger site** |  |  |
| Septum | 54 (21.3) | 13 (27.7) |
| Coronary sinus | 33 (13.0) | 9 (19.1) |
| SVC | 35 (13.8) | 4 (8.5) |
| Crista terminalis | 10 (4.0) | 0 (0.0) |
| LA posterior wall | 11 (4.3) | 3 (6.4) |
| LAA and LOM area | 12 (4.7) | 2 (4.3) |
| Bachmann bundle area | 7 (2.8) | 1 (2.1) |
| RA sinus venosa area | 11 (4.3) | 0 (0.0) |
| Low RA area | 7 (2.8) | 2 (4.3) |
| Other | 20 (7.9) | 3 (6.4) |
| Multifocal | 53 (20.9) | 10 (21.3) |

LAA, left atrial appendage; LOM, ligament of Marshall. Other abbreviations are the same as Table 1.

**Supplemental Table 2**. Type of AAD used at pre-AFCA.

|  | **Overall**  **(n=1937)** | **Extra-PV trigger (-)** | **Extra-PV trigger (+)** | **P-value** |
| --- | --- | --- | --- | --- |
|  |  |  |  | 0.107 |
| Flecainide | 860 (42.2) | 762 (42.7) | 98 (38.7) |  |
| Propafenone | 176 (8.6) | 149 (8.4) | 27 (10.7) |  |
| Amiodarone | 426 (20.9) | 383 (21.5) | 43 (17.0) |  |
| Dronedarone | 288 (14.1) | 240 (13.5) | 48 (19.0) |  |
| Sotalol | 132 (6.5) | 114 (6.4) | 18 (7.1) |  |
| Pilsicainide | 55 (2.7) | 46 (2.6) | 9 (3.6) |  |
| Not used | 99 (4.9) | 89 (5.0) | 10 (4.0) |  |

AAD, anti-arrhythmic drug. Other abbreviations are the same as Table 1.

**Supplemental Table 3**. Baseline characteristics of the study population among post-AFCA recurrence who underwent rhythm control with AADs.

|  | **Post-AFCA recurrence** | | | |
| --- | --- | --- | --- | --- |
|  | **Overall** | **Extra-PV trigger (-)** | **Extra-PV trigger (+)** | **p-value** |
| Total, n | 486 | 413 | 73 |  |
| Age | 60 (53-68) | 60 (53-68) | 60 (53-67) | 0.960 |
| Female | 161 (33.1) | 132 (32.0) | 29 (39.7) | 0.244 |
| Paroxysmal AF | 287 (59.1) | 240 (58.1) | 47 (64.4) | 0.381 |
| BMI, kg/m^2^ | 24.9 (22.9-26.9) | 25.0 (23.1-27.1) | 24.2 (22.6-26.4) | 0.048 |
| **Comorbidity** |  | | | |
| HTN | 243 (50.0) | 208 (50.4) | 35 (47.9) | 0.800 |
| DM | 81 (15.7) | 70 (16.9) | 11 (15.1) | 0.820 |
| Heart failure | 64 (13.2) | 51 (12.3) | 13 (17.8) | 0.278 |
| Vascular disease | 71 (14.6) | 65 (15.7) | 6 (8.2) | 0.134 |
| **Echocardiography parameter** |  | | | |
| EF, % | 63.0 (59.0-68.0) | 63.0 (59.0-68.0) | 64.0 (58.0-69.0) | 0.673 |
| E/e’ | 9.2 (8.0-12.0) | 9.2 (8.0-12.0) | 9.1 (8.0-11.3) | 0.634 |
| LAVI | 37.6 (30.1-47.1) | 38.0 (30.3-47.0) | 36.5 (29.2-47.4) | 0.592 |
| **Empirical linear ablation** |  | | | |
| Extra-PV LA linear line | 244 (50.2) | 200 (48.4) | 44 (60.3) | 0.082 |
| Extra-PV RA linear line | 329 (67.6) | 265 (64.2) | 54 (74.0) | 0.135 |
| **Medication** |  |  |  |  |
| RAS inhibitor | 181 (37.3) | 155 (37.6) | 26 (35.6) | 0.845 |
| Beta blocker | 153 (31.8) | 134 (32.5) | 20 (27.4) | 0.465 |
| **AAD type** |  |  |  | 0.220 |
| Flecainide | 146 (29.4) | 130 (30.5) | 16 (22.5) |  |
| Propafenone | 8 (1.6) | 8 (1.9) | 0 (0.0) |  |
| Amiodarone | 145 (29.2) | 123 (28.9) | 22 (31.0) |  |
| Dronedarone | 139 (28.0) | 119 (27.9) | 20 (28.2) |  |
| Sotalol | 56 (11.3) | 43 (10.1) | 13 (18.3) |  |
| Pilsicainide | 3 (0.6) | 3 (0.7) | 0 (0.0) |  |
| **Heart rate variability (n=374)** |  | | | |
| Mean heart rate, bpm | 71 (63-80) | 70 (63-80) | 73 (63-81) | 0.573 |
| rMSSD, ms | 17.0 (12.0-28.0) | 17.0 (12.0-28.0) | 17.0 (11.0-28.0) | 0.796 |
| LF, Hz | 7.1 (3.5-11.7) | 7.5 (3.7-11.7) | 5.1 (3.0-11.7) | 0.271 |
| HF, Hz | 6.1 (4.2-10.2) | 6.2 (4.3-10.2) | 5.8 (3.2-10.2) | 0.322 |
| LF/HF ratio | 1.0 (0.8-1.3) | 1.0 (0.8-1.4) | 1.0 (0.8-1.2) | 0.537 |

Abbreviations are the same as Table 1.

**Supplemental Table 4.** AAD responsiveness according to type of AAD used after post-AFCA recurrence.

|  | **Flecainide**  **(n=146)** | **Propafenone**  **(n=8)** | **Amiodarone**  **(n=145)** | **Dronedarone**  **(n=139)** | **Sotalol**  **(n=56)** | **Pilsicainide**  **(n=3)** | **P-value** |
| --- | --- | --- | --- | --- | --- | --- | --- |
|  |  |  |  |  |  |  | 0.301 |
| Post-AAD recurrence | 98 (67.1) | 5 (62.5) | 93 (64.1) | 78 (56.1) | 38 (67.9) | 2 (66.7) |  |

AAD, anti-arrhythmic drug. Other abbreviations are the same as Table 1.

**Supplemental Table 5**. Risk of post-AFCA recurrence and subsequent AAD responsiveness according to baseline covariates.

|  | **Post-AFCA recurrence** | | | | **Post-AAD recurrence** | | | |
| --- | --- | --- | --- | --- | --- | --- | --- | --- |
|  | **Univariable** | | **Multivariable^a^** | | **Univariable** | | **Multivariable** | |
|  | **HR (95% CI)** | **P-value** | **HR (95% CI)** | **P-value** | **HR (95% CI)** | **P-value** | **HR (95% CI)** | **P-value** |
| **Age** | 1.01 (1.00-1.02) | 0.024 | 1.00 (0.99-1.01) | 0.669 | 0.98 (0.97-0.99) | 0.003 | 0.98 (0.97-1.01) | 0.177 |
| **Male** | 0.76 (0.65-0.89) | 0.001 | 0.91 (0.76-1.09) | 0.304 | 1.29 (1.01-1.64) | 0.040 | 1.28 (0.97-1.68) | 0.077 |
| **Paroxysmal AF** | 0.57 (0.49-0.67) | <0.001 | 0.79 (0.65-0.96) | 0.020 | 0.81 (0.65-1.01) | 0.063 | 0.96 (0.72-1.27) | 0.775 |
| **HTN** | 1.18 (1.01-1.37) | 0.033 | 1.02 (0.86-1.22) | 0.806 | 0.90 (0.72-1.12) | 0.349 | 0.95 (0.73-1.24) | 0.729 |
| **BMI** | 1.01 (0.99-1.04) | 0.335 | 1.01 (0.98-1.04) | 0.510 | 1.02 (0.98-1.05) | 0.329 | 1.01 (0.97-1.05) | 0.788 |
| **DM** | 1.01 (0.82-1.25) | 0.916 | 0.97 (0.77-1.21) | 0.771 | 0.91 (0.67-1.22) | 0.512 | 0.97 (0.70-1.34) | 0.838 |
| **Heart failure** | 1.49 (1.20-1.85) | <0.001 | 1.10 (0.83-1.47) | 0.502 | 1.41 (1.02-1.94) | 0.037 | 1.14 (0.74-1.75) | 0.563 |
| **Vascular disease** | 1.02 (0.81-1.28) | 0.864 | 0.98 (0.77-1.25) | 0.876 | 0.68 (0.49-0.95) | 0.023 | 0.69 (0.48-1.01) | 0.083 |
| **EF** | 0.99 (0.98-1.00) | 0.129 | 1.01 (1.00-1.02) | 0.295 | 0.98 (0.97-0.99) | 0.001 | 0.99 (0.97-1.01) | 0.287 |
| **E/e’** | 1.02 (1.00-1.03) | 0.047 | 0.98 (0.97-1.00) | 0.127 | 1.01 (0.98-1.04) | 0.511 | 1.04 (1.01-1.08) | 0.017 |
| **LAVI** | 1.02 (1.02-1.03) | <0.001 | 1.02 (1.01-1.02) | <0.001 | 1.01 (1.00-1.01) | 0.114 | 1.00 (0.99-1.01) | 0.729 |
| **Extra-PV LA linear ablation** | 1.55 (1.33-1.80) | <0.001 | 1.00 (0.83-1.21) | 0.976 | 1.21 (0.97-1.51) | 0.095 | 1.25 (0.95-1.64) | 0.116 |
| **Extra-PV RA linear ablation** | 0.86 (0.73-1.01) | 0.063 | 0.73 (0.45-1.17) | 0.188 | 1.05 (0.83-1.32) | 0.709 | 1.30 (0.87-1.50) | 0.196 |
| **AAD after 3 month blanking period** | 3.11 (2.67-3.62) | <0.001 | 2.70 (2.28-3.19) | <0.001 | NA | | | |

AAD, anti-arrhythmic drug; CI, confidence interval; HR, hazard ratio; HRV, heart rate variability. Other abbreviations are the same as Table 1.

^a^ Multivariable Cox proportional hazard model was adjusted for age, sex, AF type, HTN, DM, BMI, heart failure, vascular disease, EF, E/e’, LAVI, Extra-PV LA linear ablation, Extra-PV RA linear ablation, and AAD after 3 months blanking period.

**Supplemental Figure 1**. Freedom from post-AFCA recurrence and subsequent AAD responsiveness according to HRV parameters.


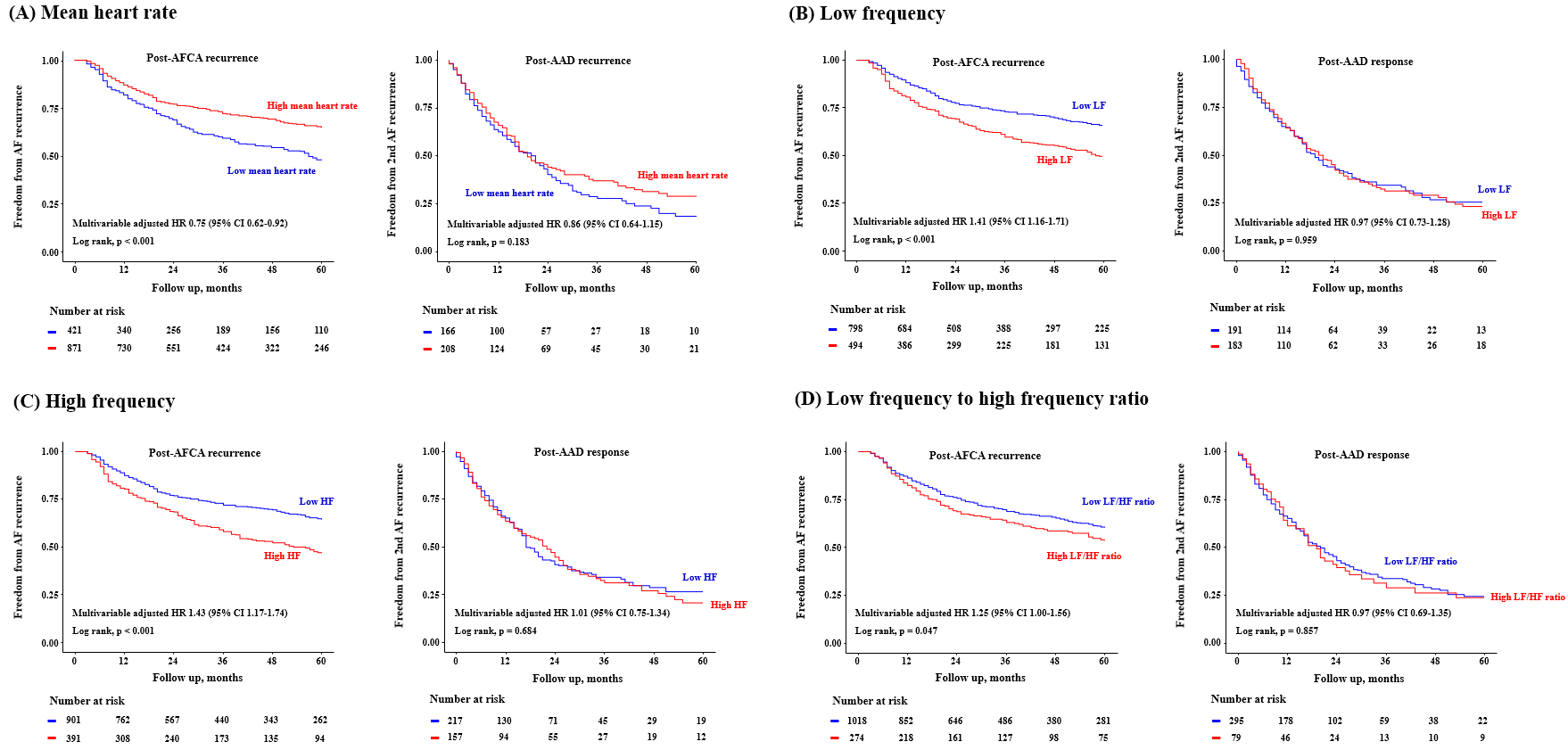


AAD, anti-arrhythmic drug; CI, confidence interval; HRV, heart rate variability; HR, hazard ratio. Other abbreviations are the same as in Table 1. The cut-off values for HRV parameters were derived from the Youden index.

**Supplemental Figure 2**. Freedom from post-AFCA recurrence and subsequent AAD responsiveness according to ExPVT and rMSSD after excluding those who underwent extra PV LA ablations.


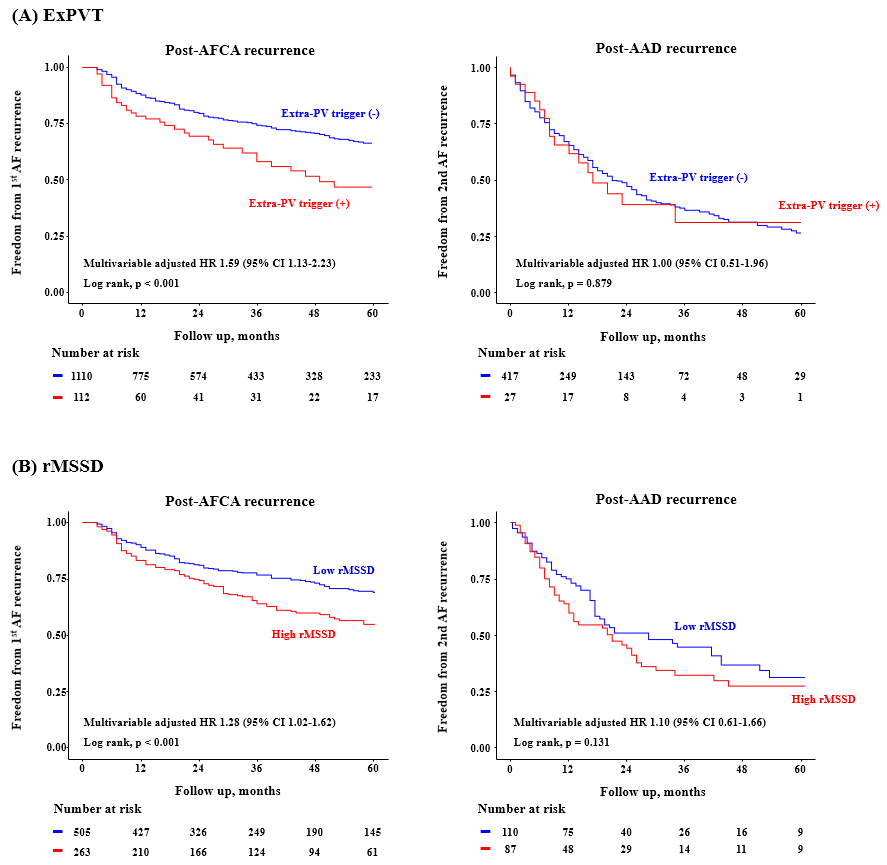


AAD, anti-arrhythmic drug; CI, confidence interval; HR, hazard ratio. Other abbreviations are the same as in Table 1. The cut-off value for rMSSD was derived from the Youden index.
